# High glucose levels boost the aggressiveness of highly metastatic cholangiocarcinoma cells via O-GlcNAcylation

**DOI:** 10.1038/srep43842

**Published:** 2017-03-06

**Authors:** Chatchai Phoomak, Kulthida Vaeteewoottacharn, Atit Silsirivanit, Charupong Saengboonmee, Wunchana Seubwai, Kanlayanee Sawanyawisuth, Chaisiri Wongkham, Sopit Wongkham

**Affiliations:** 1 Department of Biochemistry, Faculty of Medicine, Khon Kaen University, Khon Kaen, 40002, Thailand; 2 Liver Fluke and Cholangiocarcinoma Research Center, Faculty of Medicine, Khon Kaen University, Khon Kaen, 40002, Thailand; 3 Department of Forensic Medicine, Faculty of Medicine, Khon Kaen University, Khon Kaen, 40002, Thailand

## Abstract

Increased glucose utilization is a feature of cancer cells to support cell survival, proliferation, and metastasis. An association between diabetes mellitus and cancer progression was previously demonstrated in cancers including cholangiocarcinoma (CCA). This study was aimed to determine the effects of high glucose on protein O-GlcNAcylation and metastatic potentials of CCA cells. Two pairs each of the parental low metastatic and highly metastatic CCA sublines were cultured in normal (5.6 mM) or high (25 mM) glucose media. The migration and invasion abilities were determined and underlying mechanisms were explored. Results revealed that high glucose promoted migration and invasion of CCA cells that were more pronounced in the highly metastatic sublines. Concomitantly, high glucose increased global O-GlcNAcylated proteins, the expressions of vimentin, hexokinase, glucosamine-fructose-6-phosphate amidotransferase (GFAT) and O-GlcNAc transferase of CCA cells. The glucose level that promoted migration/invasion was shown to be potentiated by the induction of GFAT, O-GlcNAcylation and an increase of O-GlcNAcylated vimentin and vimentin expression. Treatment with a GFAT inhibitor reduced global O-GlcNAcylated proteins, vimentin expression, and alleviated cell migration. Altogether, these results suggested the role of high glucose enhanced CCA metastasis via modulation of O-GlcNAcylation, through the expressions of GFAT and vimentin.

Cancer cells require high glucose uptake for energy and metabolic intermediate production to support cell survival, growth and metastasis. As a consequence, a high glucose condition has been shown to promote progression in many cancer cells^1^, e.g., colon, breast, prostate, and bladder^2^,^3^,^4^. Many preclinical studies have indicated positive correlations between migration/invasion abilities of cancer cells and glucose levels as demonstrated in colon^5^ and lung cancer cells^6^. These observations may reflect the shorter survival of cancer patients with diabetes mellitus than those without diabetes^7^,^8^.

Cholangiocarcinoma (CCA) is a rare tumor worldwide but highly prevalent in Northeast Thailand. CCA is slow growing but highly metastatic, therefore, most of the patients present in an advanced stage with poor prognosis^9^,^10^. The positive linkage between diabetes mellitus and CCA in Northeast Thailand was suggested by mortality surveys^11^. Recently, the effects of high glucose in promoting cell proliferation, adhesion, migration, and invasion were demonstrated in CCA cell lines. The mechanism is partly explained by the increases of STAT3 phosphorylation and nuclear translocation, the up-regulations of cyclin D1, vimentin, and matrix metalloproteinase 2 (MMP2)^12^.

O-GlcNAcylation is a post-translational modification of protein by adding a single N-acetylglucosamine (GlcNAc) to serine or threonine by O-GlcNAc transferase (OGT). This process can be reversed by O-GlcNAcase (OGA)^13^. Normally, the majority of intracellular glucose is shunted to the glycolysis pathway and only 2–5% glucose enters the hexosamine biosynthesis pathway (HBP) to produce uridine diphospho-N-acetylglucosamine (UDP-GlcNAc), a substrate for glycosylation, e.g., O-GlcNAcylation. The rate of HBP can be regulated by the concentrations of the substrates, such as glucose and GlcNAc, or controlled by an expression of the rate limiting enzyme; glucosamine-fructose-6-phosphate amidotransfrase (GFAT)^14^,^15^. Increasing glucose uptake may promote glucose flux through HBP and subsequently increase O-GlcNAcylation. The association between an elevation of global O-GlcNAcylated proteins and tumor progression has been reported^16^. The present authors previously showed that OGT is over-expressed in CCA tissues and increased OGT is correlated with shorter survival of CCA patients^17^. Moreover, knockdown of OGT alleviates the migration/invasion of CCA cells via suppression of NF-κB nuclear translocation^18^. Nevertheless, the mechanisms by which glucose promotes O-GlcNAcylation and CCA progression remain unclear.

The present study was designed to test the crucial role of high glucose in promoting CCA cell migration/invasion, which, in fact, was found to be more pronounced in the highly metastatic cells. The tests in this study were further designed to indicate if the association between high glucose and HBP activation in CCA cells does occur, which would then subsequently increase O-GlcNAcylation and expression of vimentin, leading to the increased motility of cells. Taken together, the present study shows for the first time in the results, the implications of high glucose on HBP-modulated O-GlcNAcylation and aggressiveness of CCA cells. The findings from this study, not only fulfill the understanding of hyperglycemic conditions promoting CCA progression, but also suggest the possible use of GFAT as a new therapeutic target for CCA treatment.

## Results

### High glucose promoted migration, invasion, and epithelial-mesenchymal transition (EMT) of CCA cell lines

**Figure 1:**
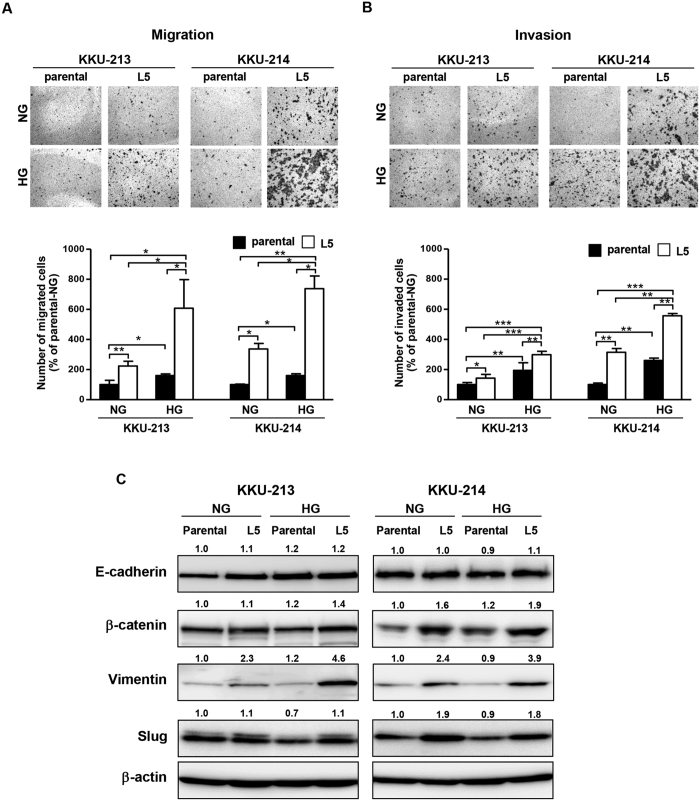
Glucose enhanced migration, invasion, and vimentin expression of highly metastatic CCA cells. The parental CCA cells (KKU-213 and KKU-214) and the highly metastatic L5 counterpart cells were cultured in normal glucose (NG) or high glucose (HG) DMEM. (**A**) migrations and (**B**) invasions were tested by the Boyden chamber assay. Numbers of migrated and invaded cells were quantitated as the percentages of the parental cells in NG. The results (mean ± SD) were the averages from two independent experiments; (**C**) The expressions of EMT markers (E-cadherin, β-catenin, vimentin, and slug) were investigated using western blotting. The intensity of each protein band was normalized by β-actin. The numbers on top of the western blot represent the relative expressions of each protein band by giving that of the parental-NG as 1. The data represent one of two independent experiments. *P < 0.05; **P < 0.01; ***P < 0.001. Full length blots of 1C are presented in Supplementary Figure S2.

Two pairs of CCA cells with different metastatic potentials, the parental low metastatic cells, KKU-213 and KKU-214, and the highly metastatic cells designated as L5, KKU-213L5 and KKU-214L5, cultured in normal and high glucose DMEM, were used to investigate the migration and invasion abilities using the Boyden chamber assay. As shown in Fig. 1A, the migrated cell numbers of L5 of both cell lines were significantly higher than those of their parental cells in both normal and high glucose conditions. In addition, high glucose potentiated the migration ability of both parental and L5 cells. High glucose, however, had a more pronounced effect on the L5 cells; approximately 2–3 fold for the L5 cells and 1.5 fold for the parental cells. Similar results were observed for the invasion abilities (Fig. 1B).

Since the effect of high glucose was more obvious for the migration, therefore, the expressions of EMT markers were elucidated. As shown in Fig. 1C, by giving the expression of EMT markers from the parental cells in normal glucose reference as 1, the expressions of epithelial marker, E-cadherin, and the mesenchymal marker, slug, of the parental and L5 cells seemed to be unaltered in the high glucose condition. The expression levels of mesenchymal markers, β-catenin and vimentin, of KKU-213, KKU-214, and their L5 sublines, however, were increased in the high glucose condition. The expression levels of vimentin were of interest because these corresponded with the glucose levels and were consistently observed in both cell lines. Vimentin was increased, 2 fold in KKU-213L5 and 1.6 fold in KKU-214L5.

### High glucose increased O-GlcNAcylation in L5 cells more than in the parental cells

**Figure 2:**
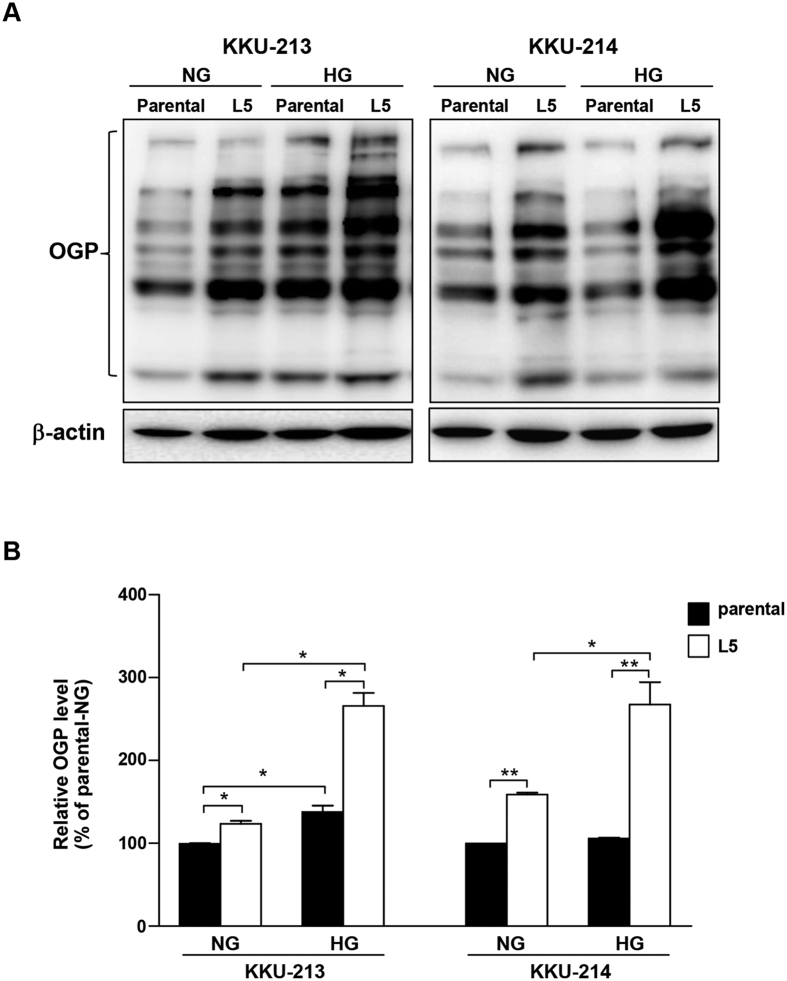
High glucose induced global O-GlcNAcylation of CCA cells. The O-GlcNAcylated protein (OGP) levels were determined using western blotting; (**A**) OGP western blot; (**B**) quantitative analyses of OGP normalized by β-actin and giving that of parental cells cultured in normal glucose (NG) as 1. The data represent one of two independent experiments. *P < 0.05; **P < 0.01. HG = high glucose. Full length blots of 2A are presented in Supplementary Figure S3.

To reveal the effect of glucose on O-GlcNAcylation, the O-GlcNAcylated protein levels of the parental and L5 cells cultured in the normal and high glucose conditions were examined. The global O-GlcNAcylated proteins of the L5 cells were higher than those of the corresponding parental cells in both normal and high glucose conditions (Fig. 2A,B). Moreover, the effect of glucose-promoting O-GlcNAcylation was more prominent in the L5 cells than the parental cells; 2.5 fold in KKU-213L5 and 1.5 fold in KKU-214L5 (Fig. 2B).

### High glucose increased O-GlcNAcylated vimentin and vimentin stability

**Figure 3:**
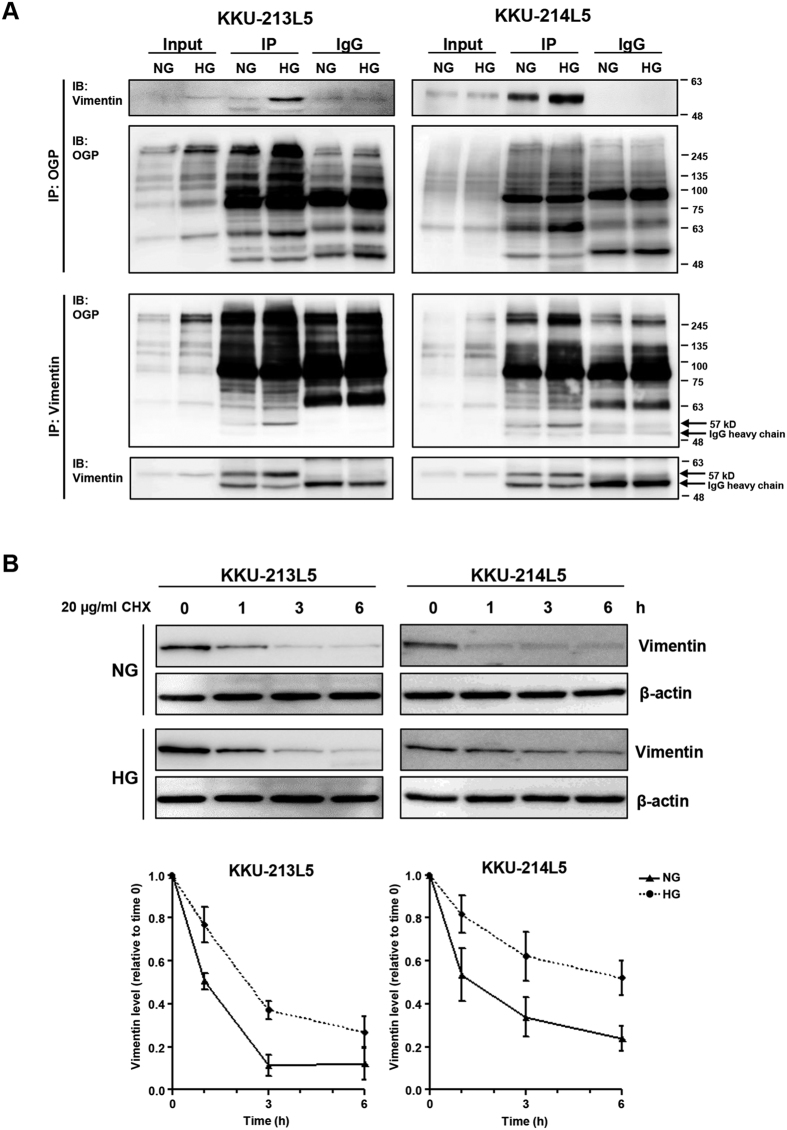
Glucose increased O-GlcNAcylation and stability of vimentin. (**A**) Cell lysates were immunoprecipitated with anti-O-GlcNAcylated proteins (OGP) or anti-vimentin followed by western blotting of vimentin and OGP. (**B**) L5 cells in normal glucose (NG) or high glucose (HG) were treated with 20 μM of CHX for 0, 1, 3, and 6 h, and vimentin levels were compared between L5 cells in NG and HG. The levels of vimentin were quantitated by assigning those at 0 time as 1. The data are presented as mean ± SEM from two independent experiments. Full length blots of 3A and 3B are presented in Supplementary Figures S4 and S5.

As expressions of vimentin and O-GlcNAcylation were obviously elevated in L5 cells cultured in the high glucose, the linkage between vimentin and O-GlcNAcylation was investigated next. Cell lysates of L5 cells cultured in the normal and high glucose media were subjected to immunoprecipitation using anti-O-GlcNAcylated protein. As shown in Fig. 3A (upper panel), O-GlcNAcylated vimentin was increased in L5 cells cultured in high glucose. The similar results were obtained in the reversed-immunoprecipitation using anti-vimentin (Fig. 3A, lower panel). To ensure the O-GlcNAcylation of vimentin, the succinylated wheat germ agglutinin (sWGA) lectin pull-down assay was performed as previously described^19^ in KKU-213L5. The result showed that O-GlcNAcylated vimentin was increased in high glucose treated cells. The specific interaction of O-GlcNAcylated vimentin and sWGA were assured by the neutralization of sWGA with GlcNAc (Supplementary Fig. S1). The signals of O-GlcNAcylated vimentin, sWGA-conjugated proteins and O-GlcNAcylated proteins were diminished in the presence of GlcNAc. These results affirmed the increased O-GlcNAcylated vimentin in high glucose treated cells. Since stability of protein can be modulated via O-GlcNAcylation, therefore, whether the increase of O-GlcNAcylated vimentin in L5 under high glucose conditions was due to the increases of vimentin stability was determined. The L5 cells were treated with cycloheximide (CHX) at different time points and vimentin expressions were measured. As shown in Fig. 3B, the levels of vimentin of both L5 sublines cultured in high glucose were reduced at a slower rate than those cultured in normal glucose.

### Glucose enhanced HK-II, GFAT, and OGT expressions in L5 cells

**Figure 4:**
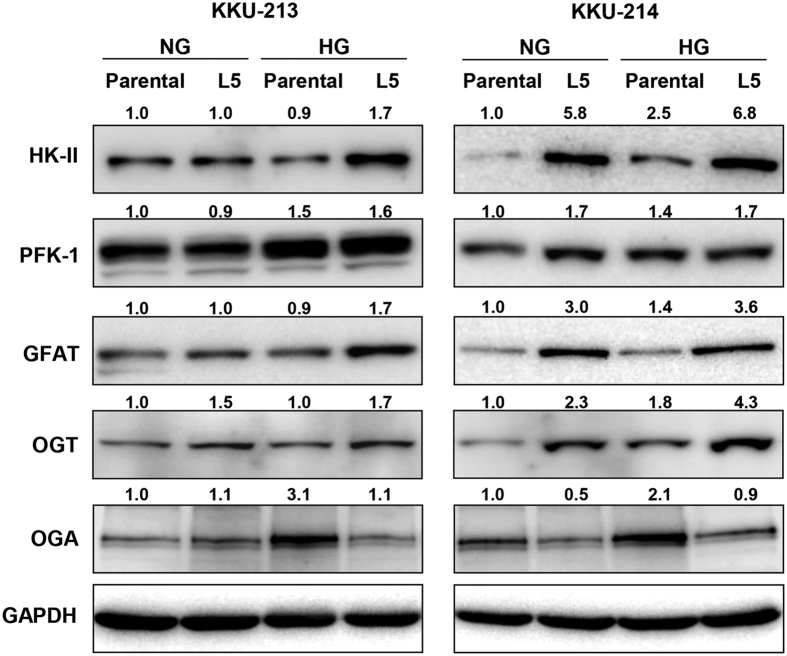
High glucose induced expressions of key regulatory enzymes involved in O-GlcNAcylation. The expressions of HK-II, PFK-1, GFAT, OGT and OGA of the parental and highly metastatic L5 cells cultured in normal glucose (NG) and high glucose (HG) were compared using western blotting. The intensity of each protein band was normalized by GAPDH. The numbers on top of the western blot represent the relative expressions of each protein by giving the parental-NG as 1. The data represent one of two independent experiments. Full length blots are presented in Supplementary Figure S6.

As O-GlcNAcylation can be modulated by glycolysis, HBP, and O-GlcNAc cycling, the effects of glucose on the expressions of the regulatory enzymes in glycolysis (hexokinase-II; HK-II, phosphofructokinase-1; PFK-1), HBP (GFAT) and O-GlcNAc cycling (OGT and OGA) in L5 cells were examined. As shown in Fig. 4, by giving the enzyme expressions of the parental cells in normal glucose as 1, KKU-213L5 in normal glucose had higher levels of OGT than the parental cells, whereas, levels of HK-II, PFK-1, and GFAT were similar. High glucose induced expressions of HK-II, PFK-1, and GFAT enzymes in KKU-213L5 cells but not in the parental cells. In contrast, KKU-214L5 in normal glucose had higher expressions of HK-II, PFK-1, GFAT and OGT than its parental cells. KKU-214 cells cultured in high glucose increased expressions of these enzymes in both L5 and parental cells when compared with the corresponding cells in normal glucose. Increased expression of OGA, however, was observed only in the parental cells of both cell lines when cultured in high glucose. In summary, under the high glucose conditions, increased levels of HK-II, GFAT, and OGT were observed in the highly metastatic L5 cells while increases of OGA were found only in the parental cells.

### The GFAT inhibitor and si-OGT treatment suppressed glucose-induced O-GlcNAcylation, cell migration, and vimentin expression in L5 cells

**Figure 5:**
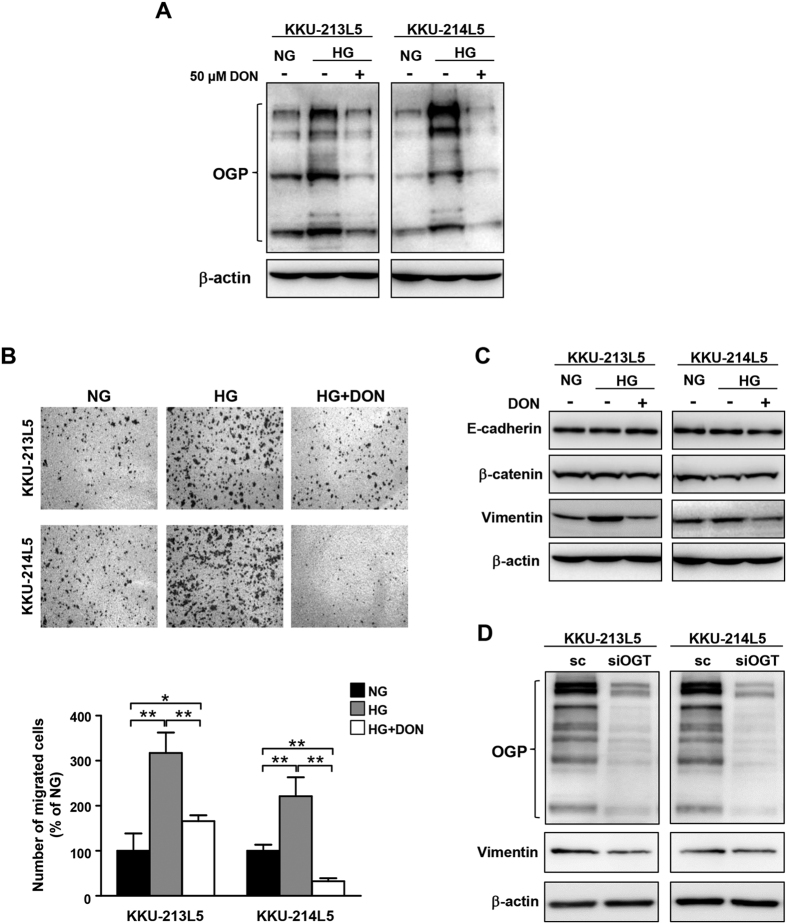
The GFAT inhibitor and si-OGT treatment suppressed glucose-induced O-GlcNAcylation, cell migration, and vimentin expression in L5 cells. L5 cells in high glucose (HG) were treated with DON, a GFAT inhibitor, for 24 h before subjecting to the migration assay and western blotting. L5 cells in normal glucose (NG) were used as references. (**A**) O-GlcNAcylated protein (OGP) levels, (**B**) cell migration and (**C**) vimentin expressions were determined. (**D**) L5 cells were treated with siOGT for 48 h and then were used for OGP and vimentin determination. β-actin was used as a protein loading control. Numbers of migrated cells were quantitated as the percentage of the parental cells in NG. The data represent one of two independent experiments. *P < 0.05; **P < 0.01. Full length blots of 5A, 5C, 5D are presented in Supplementary Figure S7.

As the increased expression of GFAT by high glucose was consistently observed in L5 of both cell lines, the significances of GFAT in mediating O-GlcNAcylation and migration of L5 cells were next examined. GFAT is a key enzyme involved in the synthesis of UDP-GlcNAc, a substrate of O-GlcNAcylation; alteration of GFAT activity might affect cellular O-GlcNAcylation and the migration of CCA cells. To test this, 6-diazo-5-oxo-l-norleucine (DON), a competitive inhibitor of GFAT was applied. Using L5 in normal glucose without DON as the reference, 50 μM DON treated L5 cells in high glucose reduced O-GlcNAcylated proteins to the basal levels of L5 cultured in normal glucose (Fig. 5A). The results were consistently observed in both L5 cells. Suppression of GFAT activity by DON also significantly decreased the motility of both L5 cells in high glucose, by 50% in KKU-213L5 and by 85% in KKU-214L5 (Fig. 5B). Treated cells with DON significantly suppressed the expression of vimentin but not those of E-cadherin and β-catenin (Fig. 5C). Moreover, the association of O-GlcNAcylation and vimentin expression was investigated in si-OGT treated L5 cells in high glucose condition. As shown in Fig. 5D, decreased OGT lessened the amount of O-GlcNAcylated proteins and vimentin expression in both L5 cells. The result supported the association of O-GlcNAcylation and vimentin protein stability regardless of high glucose in the cultured media.

### GFAT expression in CCA tissues of patients was correlated with O-GlcNAcylated protein level

**Figure 6:**
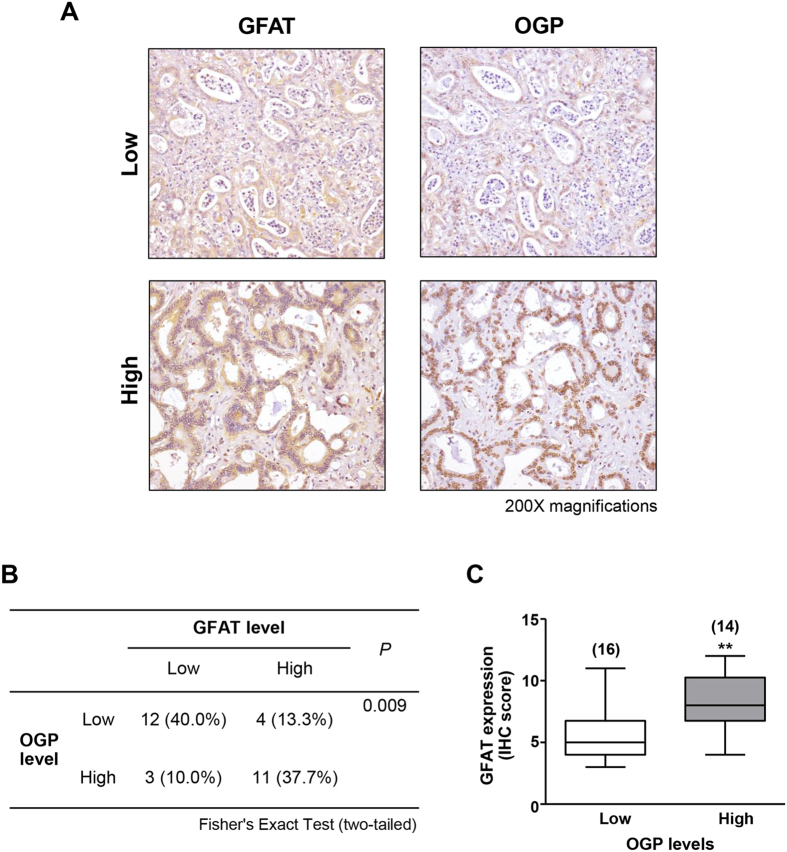
GFAT expressions in CCA tissues from patients correlated with O-GlcNAcylated protein (OGP) levels. (**A**) IHC staining of GFAT and OGP in two representative pairs of CCA tissues. (**B**) The correlations between GFAT and OGP expressions were analyzed by Fisher’s Exact test (N = 30). (**C**) Mean expression of GFAT of the low OGP group was significantly lower than that of the high OGP group (**P < 0.01; Mann Whitney test). The numbers of samples in each group are indicated.

Because GFAT expression had a positive relationship with the O-GlcNAcylated protein level of CCA cells, whether or not this association existed in CCA tissues of the patients was next examined. The levels of GFAT and O-GlcNAcylated proteins were investigated in CCA tissues using immunohistochemistry (IHC) and quantitated as IHC scores. Expressions of GFAT and O-GlcNAcylated proteins were divided into low- and high-expressed groups using the median IHC scores. The positive correlations between GFAT expression and O-GlcNAcylated protein levels were observed (Fig. 6A and B, Fisher’s Exact test, *P* = 0.009). CCA patients (37.7%), who had high GFAT expression in tumor tissues, also had high O-GlcNAcylated protein levels and patients (40%), whose tissues had low GFAT expression, also had low levels of O-GlcNAcylated protein. In addition, expression levels of GFAT from the patients with low O-GlcNAcylated proteins were significantly lower than those with high O-GlcNAcylated proteins (Fig. 6C, Mann-Whitney test, *P* = 0.0085).

## Discussion

EMT is an important step in cancer metastasis. Several studies reported the significance of glucose in promoting EMT and metastasis of cancer. Currently, the linkage of high glucose enhancing metastasis of CCA has been emphasized^12^. In this study, it was demonstrated for the first time that high glucose had a more pronounced effect on the highly metastatic CCA cells than the low metastatic counterparts. The molecular mechanism was shown to be via the up-regulation of GFAT in the HBP which in turn increased the O-GlcNAcylation of proteins, especially vimentin. O-GlcNAcylation of vimentin may increase vimentin stability and consequently promote migration and invasion of CCA cells. The impact of GFAT on O-GlcNAcylation in CCA was emphasized by the positive correlation between the expression of GFAT and O-GlcNAcylated protein levels found in the tumor tissues of CCA patients. These findings indicate the significance of high glucose on promoting O-GlcNAcylation and cancer aggressiveness, especially of the highly metastatic CCA cells. Hence, GFAT might be a potential target for CCA treatment, particularly in patients with hyperglycemia or diabetes mellitus.

Several cancer cells increase glucose uptake to provide energy and metabolic intermediates to support cell survival, proliferation, and metastasis^1^. In this study, high glucose induced metastatic phenotypes of the parental and the highly metastatic derivatives, L5 cells. The highly metastatic cells seemed to respond to high glucose more aggressively than the lower metastatic parental cells. This observation was concomitant with higher O-GlcNAcylation found in the L5 cells. The connection between O-GlcNAcylation and aggressive phenotypes under the stimulation of glucose have been reported in many cancers^14^,^20^,^21^,^22^,^23^, however, the obvious effect of glucose on highly metastatic cells was first observed in this study.

Addition or removal of O-GlcNAc modulates protein functions in many ways, e.g., regulating protein phosphorylation, altering the stability/localization of proteins, and modulating protein-protein interactions^15^,^24^. In the present study, promoting cell migration of the highly metastatic L5 cells by high glucose was concomitantly observed with the induction of vimentin O-GlcNAcylation and with a slower decline of vimentin level in the presence of cycloheximide. Hence, the O-GlcNAcylation of vimentin may promote protein stability and consequently stimulate cell migration. Under high glucose stimulation, over-expression of vimentin was observed in the L5 cells. High glucose promoted O-GlcNAcylation and the stability of vimentin was demonstrated by this study. The significance of O-GlcNAcylation on the stability of proteins, for instance, c-Myc and FOXM1, has been reported^25^,^26^. In the present study, the stability of vimentin in L5 cells under high glucose was prolonged when compares to those under normal glucose. The O-GlcNAcylation of vimentin was previously reported using mass spectrometry^27^ and immunoprecipitation^28^, however, the effects of glucose on O-GlcNAcylation and stability of vimentin were reported for the first time in this study.

**Figure 7:**
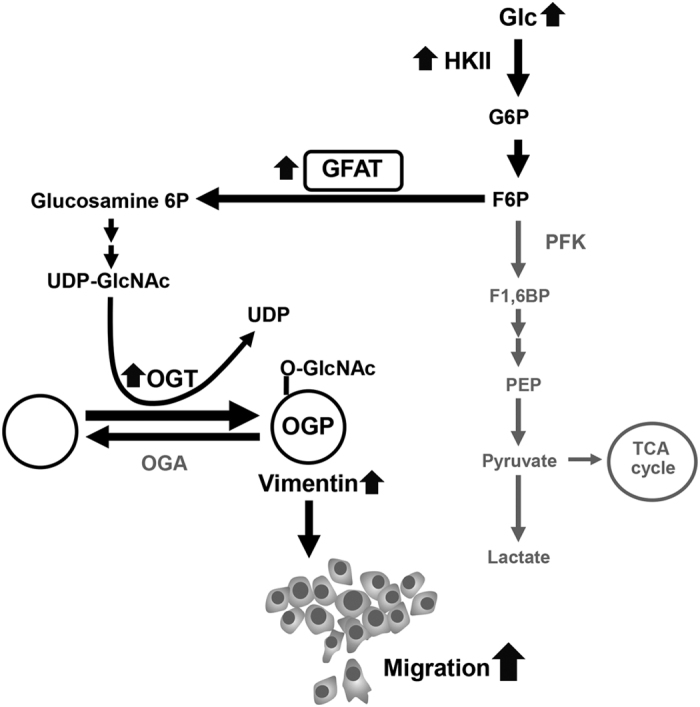
Schematic diagram represents the mechanism of high glucose promoting cell migration via O-GlcNAcylation. High glucose induces high expressions of HK-II, GFAT, and OGT. HK-II catalyzes glucose to glucose-6-phosphate (G6P) and then fructose-6-phosphate (F6P) which can be fluxed into HBP via GFAT action. Elevations of OGT and UDP-GlcNAc, GFAT product, promote cellular O-GlcNAcylation. As a result, vimentin is O-GlcNAcylated. Consequently, O-GlcNAcylated vimentin may increase its stability and promotes CCA cell motility.

Glycolysis, HBP and O-GlcNAc cycling directly and indirectly modulate protein O-GlcNAcylation (Fig. 7). The molecular linkage between high glucose and the expressions of key enzymes in these pathways in CCA cells were explored. The interplay between these pathways to support higher O-GlcNAcylation of L5 was obvious in KKU-214L5 when compared to KKU-214 in normal glucose. The increases of HK-II, PFK-1, GFAT and OGT with decreased OGA were observed in KKU-214L5 compared to those of the parental cells. In contrast, the parental cells, KKU-213, in normal glucose possessed high levels of HK-II, PFK-1 and GFAT, and hence, the expression of these enzymes did not increase in the L5 cells. In the normal glucose condition, the level of OGT seemed to be responsible for the higher O-GlcNAcylation in L5 cells of both CCA cell lines. Under the high glucose condition, high glucose stimulated the expression of all enzymes tested in KKU-213L5, KKU-214 and KKU-214L5. High glucose could stimulate only the expressions of PFK-1 and OGA of the parental KKU-213. Altogether, high glucose could promote O-GlcNAcylation in L5 cells via up-regulation of the key enzymes involved in O-GlcNAcylation, namely, HK-II, GFAT and OGT, whereas OGA, the enzyme that removes O-GlcNAc was unaltered. Consistent with the current study, the increased global protein O-GlcNAcylation by shunting glucose to HBP was reported in TGF-β-stimulated A549, lung cancer cells^29^. The increased UDP-GlcNAc production drives the O-GlcNAcylations of proteins through the up-regulation of OGT and the induction of EMT process thereafter. The glucose bypassing is modulated by the activation of GFAT expression. Even though there are differences in the stimulants (TGF-β vs. high glucose in this study), the commonality of GFAT promoting protein O-GlcNAcylation and EMT stimulation is evidence.

It is worth mentioning here that high glucose stimulated the expression of OGA in the parental cells of both cell lines. Under the stress of high glucose, the parental cells could possibly respond to reduce the O-GlcNAcylation by increasing OGA; in contrast, the highly metastatic cells were prone to increase O-GlcNAcylation by increasing the expressions of GFAT and OGT. The molecular mechanism by which the parental cells and the highly metastatic cells regulate cellular O-GlcNAcylation is of interest but it is, however, beyond the scope of this study.

The association of OGT and migration of CCA cells was reported previously^18^. In the present study, the role of GFAT in the increase of O-GlcNAcylation and migration in L5 cells under high glucose condition was shown to be significant. Suppression of GFAT action using DON, a GFAT inhibitor, significantly reduced O-GlcNAcylation and migration of L5 cells in both CCA cell lines. As a consequence, vimentin was remarkably reduced in DON treated cells. The use of DON for cancer treatment has been reported in a series of experiments. DON could inhibit cancer growth and systemic metastasis in the VM-M3 murine model^30^. In breast (MCF7) and colorectal (HT29) cancer cell lines, DON and azaserine treatments reduced cell proliferation via the inhibition of O-GlcNAcylation and the reduced stability of β-catenin^31^. Similar effects were reported in endometrial cancer cells, AN3CA and HEC-1-B^32^. Nonetheless, the effect of glucose induced β-catenin expression was not observed in CCA cells in the current study. The association of GFAT and O-GlcNAcylation in CCA patients’ tissues was further highlighted herein. High expression of GFAT was correlated with high O-GlcNAcylated protein levels in tumor tissues of CCA patients. All the evidence in the preclinical studies suggested GFAT is a potential target for CCA treatment. Both DON and azaserine, however, have been evaluated in clinical trials and showed adverse effects with variable degrees of gastrointestinal toxicity, myelo-suppression, and neurotoxicity^33^,^34^. A new potent GFAT inhibitor with less toxicity is challenging for cancer therapy. The *in vivo* studies using a GFAT inhibitor on growth and metastasis of CCA should be explored.

As summarized in Fig. 7, high glucose induced progression of CCA cells was prominent in the highly metastatic cells. The mechanism was demonstrated partly via the increase of cellular O-GlcNAcylation. GFAT was demonstrated to play a significant role in high glucose induced O-GlcNAcylation and cell migration particularly in the highly metastatic L5 cells. Vimentin was one glucose induced protein in which the stability of this protein may be prolonged, perhaps via GFAT and O-GlcNAcylation. These processes might finally potentiate EMT change and increase cell motility. The association of GFAT and O-GlcNAcylated protein was emphasized not only in the *in vitro* study but also in the tumor tissues of CCA patients. Altogether, the adverse effects of high glucose on aggressiveness of CCA cells, especially on the highly metastatic cells, was emphasized. The potential role of GFAT as a new therapeutic target, especially for advanced CCA, is suggested. Attempts to control blood glucose and/or reducing GFAT action in CCA patients may minimize the effects of high glucose on the aggressiveness of CCA and may be of benefit in the treatment of CCA patients with diabetes mellitus. The research for a new GFAT inhibitor with safer implications requires urgent attention.

## Materials and Methods

### Reagents and antibodies

DON, O-(2-Acetamido-2-deoxy-D-glucopyranosylidenamino) N-phenylcarbamate (PUGNAc), and cycloheximide were obtained from Sigma-Aldrich (St. Louis, MO). Antibodies were purchased from various sources: anti-β-catenin (14) and anti-E-caherin (36) from BD biosciences; anti-slug (C19G7) and anti-vimentin (D21H3, for western blot) from Cell Signaling (Danvers, MA); anti-O-GlcNAc (RL-2) from Pierce Biotechnology (IL, USA); anti-GFAT (H-49), anti-HK-II (C-14), anti-OGT (F-12), and anti-PFK-1 (H-55) from Santa Cruz Biotechnology (Santa Cruz, CA); anti-OGA and control IgG for immunoprecipitation from Sigma-Aldrich (St. Louis, MO); anti-vimentin (RV202) for immunoprecipitation from Abcam (Cambridge, UK). Detail of all antibodies are listed in Supplementary Table S1.

### Cell culture

CCA cell lines (KKU-213 and KKU-214), were established from primary tumors of Thai CCA patients by Prof. B. Sripa (Faculty of Medicine, Khon Kaen University, Thailand) as previously described^35^. Cells were obtained from the Japanese Collection of Research Bioresources (JCRB) Cell Bank, Osaka, Japan. The highly metastatic CCA sublines; KKU-213L5 and KKU-214L5 were established from the parental cells, KKU-213 and KKU-214, as described elsewhere^36^.

All cell lines were cultured in Dulbecco’s Modified Eagle Medium (DMEM, Gibco, NY) containing either normal (5.6 mM glucose) or high glucose (25 mM glucose), supplemented with 10% fetal bovine serum (Gibco) and 1% antibiotic-antimycotic (Gibco), for at least 10 passages. The cultures were maintained at 37 °C, 5% CO_2_.

### CCA tissues

The 30 formalin-fixed paraffin-embed CCA tissues were obtained from the specimen bank of the Liver Fluke and Cholangiocarcinoma Research Center, Faculty of Medicine Khon Kaen University. The study protocol was approved by the Ethics Committee for Human Research, Khon Kaen University (HE581369). All cancer tissues were histologically proven as intrahepatic CCA.

### Cycloheximide treatment

Cycloheximide (CHX), a protein synthesis inhibitor, was used to determine the protein stability. CCA cells (3 × 10^5^ cells/well) were plated into a 6-well plate and incubated for 24 h and then treated with 20 μg/ml of CHX for the indicated time.

### DON treatment

DON, the competitive GFAT inhibitor, was used to determine the functional significance of GFAT. Cells were cultured in high glucose DMEM with or without 50 μM DON for 24 h before further experiments.

### Suppression of OGT expression by specific-siRNA

Specific si-OGT was used to evaluate the functional significance of vimentin O-GlcNAcylation in CCA cells. CCA cells (2 × 10^5^ cells/well) were cultured in a 6-well plate for 24 h and then cells were treated with Lipofectamine 2000 (2 μg/ml) containing 100 pmole of siOGT (Invitrogen, NY) according to the manufacturer recommendation. At 6 h after transfection, media was replaced by DMEM containing 10% FBS and cells were cultured for 48 h prior protein lysate preparation for western blotting. Control experiments were performed simultaneously; cells were treated with siControl (Negative Control siRNA, 1027310, Qiagen). The sequences of si-OGT were reported elsewhere^37^.

### Cell migration and invasion

CCA cells (3 × 10^4^ cells) were added into an upper chamber of an 8.0 μm pore size transwell (Corning Incorporated, Corning, NY) and incubated for 9 h for KKU-213/KKU-213L5 cells and 24 h for KKU-214/KKU-214L5 cells. The migrated cells were stained by 0.4% sulforhodamine B in 0.1% acetic acid. The migrated cell numbers were determined from at least 5 microscopic fields (100× magnification). For the invasion assay, the transwell was coated with 0.4 mg/ml Matrigel^TM^ (BD biosciences) and the experiment was performed as before for the migration assay. All samples were done in duplicate.

### Immunoprecipitation

Immunoprecipitation was performed as previously described^18^. Briefly, total cell lysates were incubated with anti-O-GlcNAc, anti-vimentin, or control IgG. The antibody was immobilized using protein-G sepharose beads (GE Healthcare, Buckinghamshire, UK). After centrifugation, precipitated-beads were washed, eluted, and subjected to SDS-PAGE and western blotting.

### Western blot

CCA cells were lysed in NP-40 lysis buffer^18^. Protein concentration was determined using the Bradford reagent (Bio-rad laboratories, Hercules, CA). Protein lysate was separated on SDS-PAGE and transferred onto a PVDF membrane. The immunoreactivity was detected using the ECL^TM^ Prime Western Blotting Detection System (GE Healthcare). The signals were captured by the ImageQuant™ LAS 4000 mini-image analyzer and the signal intensities were analyzed by ImageQuant™ TL analysis software (GE Healthcare).

### Immunohistochemistry of GFAT and O-GlcNAcylated proteins

Expressions of GFAT and O-GlcNAcylated proteins were determined using immunohistochemistry (IHC) staining as the standard protocol. The signals were amplified using the EnVision-system-HRP (Dako, Glostrup, Denmark). The immunoreactivity signals were developed using diaminobenzidine (Sigma-Aldrich). The IHC score was determined using Fromowitz standard as described previously^17^. The positive frequencies were scored as 0 = 0–5%; 1+ = 6–25%; 2+ = 26–50%; 3+ = 51–75%; 4+ = 75%; the positive signal intensities: 0 = no staining; 1 = light yellow; 2 = brown; 3 = dark brown; and IHC score = frequency score plus intensity score. Two independent assessors assessed IHC score without knowing information of the clinical parameters.

### Statistical analysis

The statistical significance was determined by Student’s *t*-test using GraphPad Prism^®^ 5.0 software (GraphPad software, Inc., La Jolla, CA). The correlation between the expression levels of GFAT and O-GlcNAcylated proteins in CCA tissues was analyzed using Fisher’s Exact test and Mann-Whitney test, IBM SPSS Statistics 22 software (SPSS, Chicago, IL). *P* < 0.05 was considered statistically significant.

## Additional Information

**How to cite this article:** Phoomak, C. *et al*. High glucose levels boost the aggressiveness of highly metastatic cholangiocarcinoma cells via O-GlcNAcylation. *Sci. Rep.* **7**, 43842; doi: 10.1038/srep43842 (2017).

**Publisher's note:** Springer Nature remains neutral with regard to jurisdictional claims in published maps and institutional affiliations.

## Supplementary Material

Supplementary Figures
