# Supplementary material for: High glucose levels boost the aggressiveness of highly metastatic cholangiocarcinoma cells via O-GlcNAcylation
Source: Sci Rep. 2017 Mar 6;7:43842. doi: 10.1038/srep43842 (PMC5338328; doi:10.1038/srep43842)
Supplement: Supplementary Figures [file srep43842-s1.pdf]

# High glucose levels boost the aggressiveness of highly metastatic cholangiocarcinoma cells via O-GlcNAcylation

Chatchai Phoomak[1,3], Kulthida Vaeteewoottacharn[1,3]*, Atit Silsirivanit[1,3], Charupong Saengboonmee[1,3], Wunchana Seubwai[2,3], Kanlayanee Sawanyawisuth[1,3], Chaisiri Wongkham[1,3], Sopit Wongkham[1,3]*

[1]Department of Biochemistry, Faculty of Medicine, Khon Kaen University, Khon Kaen, 40002, Thailand.

[2]Department of Forensic Medicine, Faculty of Medicine, Khon Kaen University, Khon Kaen, 40002, Thailand.

[3]Liver Fluke and Cholangiocarcinoma Research Center, Faculty of Medicine, Khon Kaen University, Khon Kaen, 40002, Thailand.

**Corresponding authors:**

Kulthida Vaeteewoottacharn, Ph.D., M.D.
Department of Biochemistry,
Faculty of Medicine, Khon Kaen University,
Khon Kaen, 40002, Thailand
Tel/Fax: +66-43-348-386
Email: kulthidava@kku.ac.th

AND Sopit Wongkham, Ph.D.
Department of Biochemistry,
Faculty of Medicine, Khon Kaen University,
Khon Kaen, 40002, Thailand
Tel/Fax: +66-43-348-386
Email: sopit@kku.ac.th

## Supplementary Materials and Methods

### Succinylated wheat germ agglutinin (sWGA) pull-down assay

The sWGA pull-down assay was performed as previously described[1]. In brief, 480 µg of cell lysates of KKU-213L5 were incubated with 40 µl of agarose-conjugated sWGA (Vector Laboratories, Burlingame, CA) with or without 0.25 M GlcNAc for overnight at 4°C. Precipitates were washed four times with NET lysis buffer (150 mM NaCl, 50 mM Tris, pH 7.4, 1 mM EDTA, 0.5% Nonidet P-40) and proteins were eluted by boiling in SDS sample buffer.

Reference:

1 Kang, J. G. *et al.* O-GlcNAc protein modification in cancer cells increases in response to glucose deprivation through glycogen degradation. *J Biol Chem* 284, 34777-34784 (2009).

**Supplementary data**

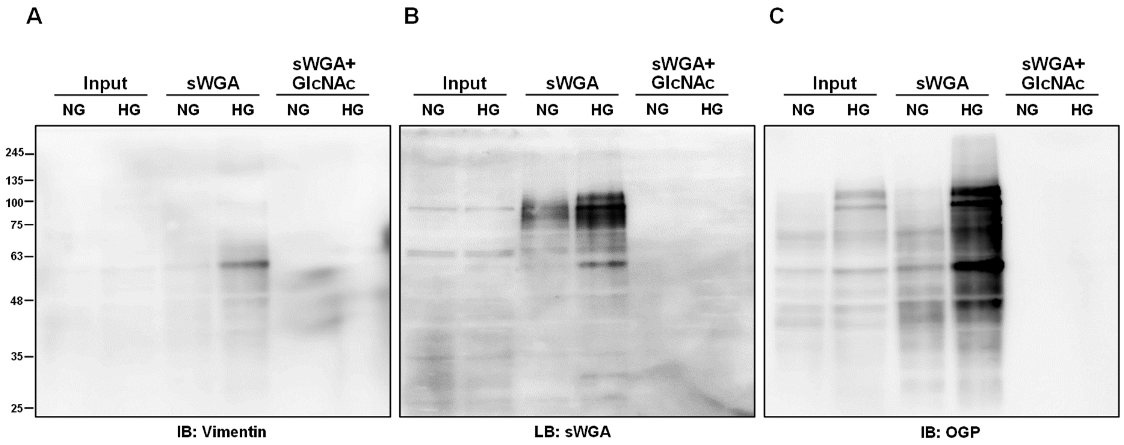


**Supplementary Figure S1. High glucose induced vimentin O-GlcNAcylation determined by sWGA lectin pull-down assay.** Total cell lysates were prepared from KKU-213L5 cells cultured in normal glucose (NG) and high glucose (HG) and were incubated with sWGA lectin agarose beads. sWGA pull-down products were immunoblotted with A) anti-vimentin, B) sWGA, and C) anti-O-GlcNAcylated proteins. LB = lectin blot.

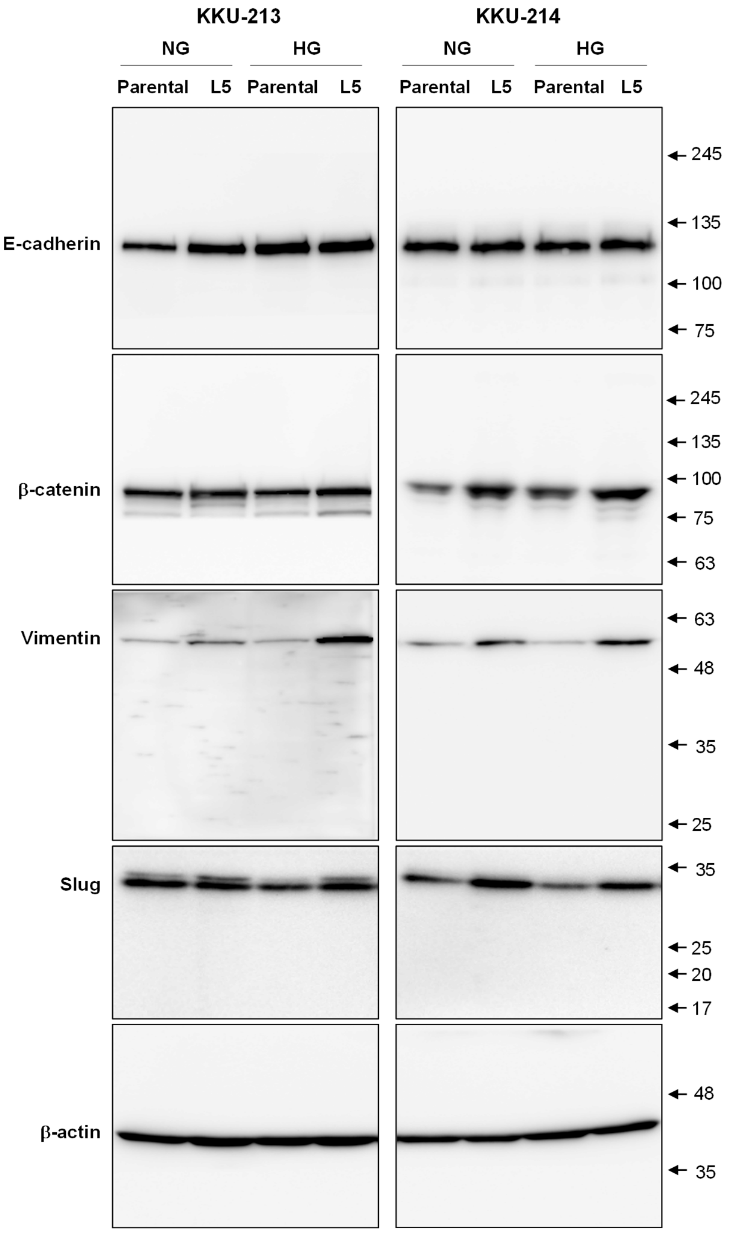


**Supplementary Figure S2. Full-length blots presented in Figure 1C.**

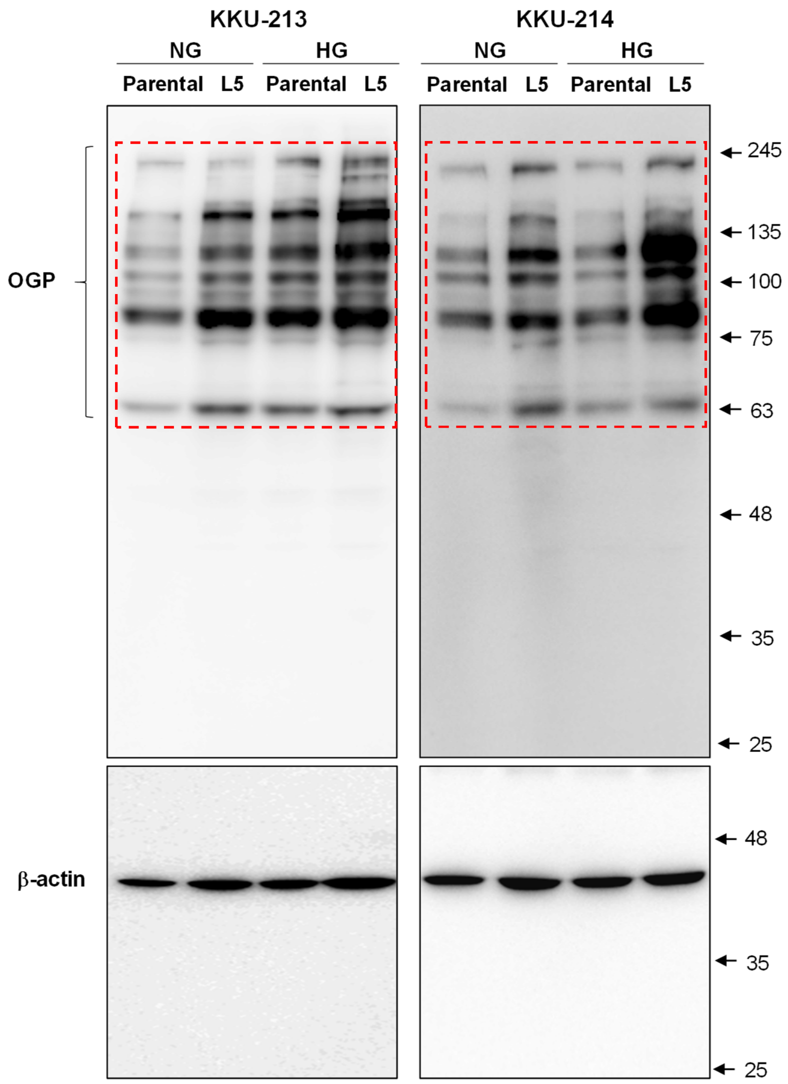


**Supplementary Figure S3. Full-length blots presented in Figure 2A.** Red boxes represented the selected areas.

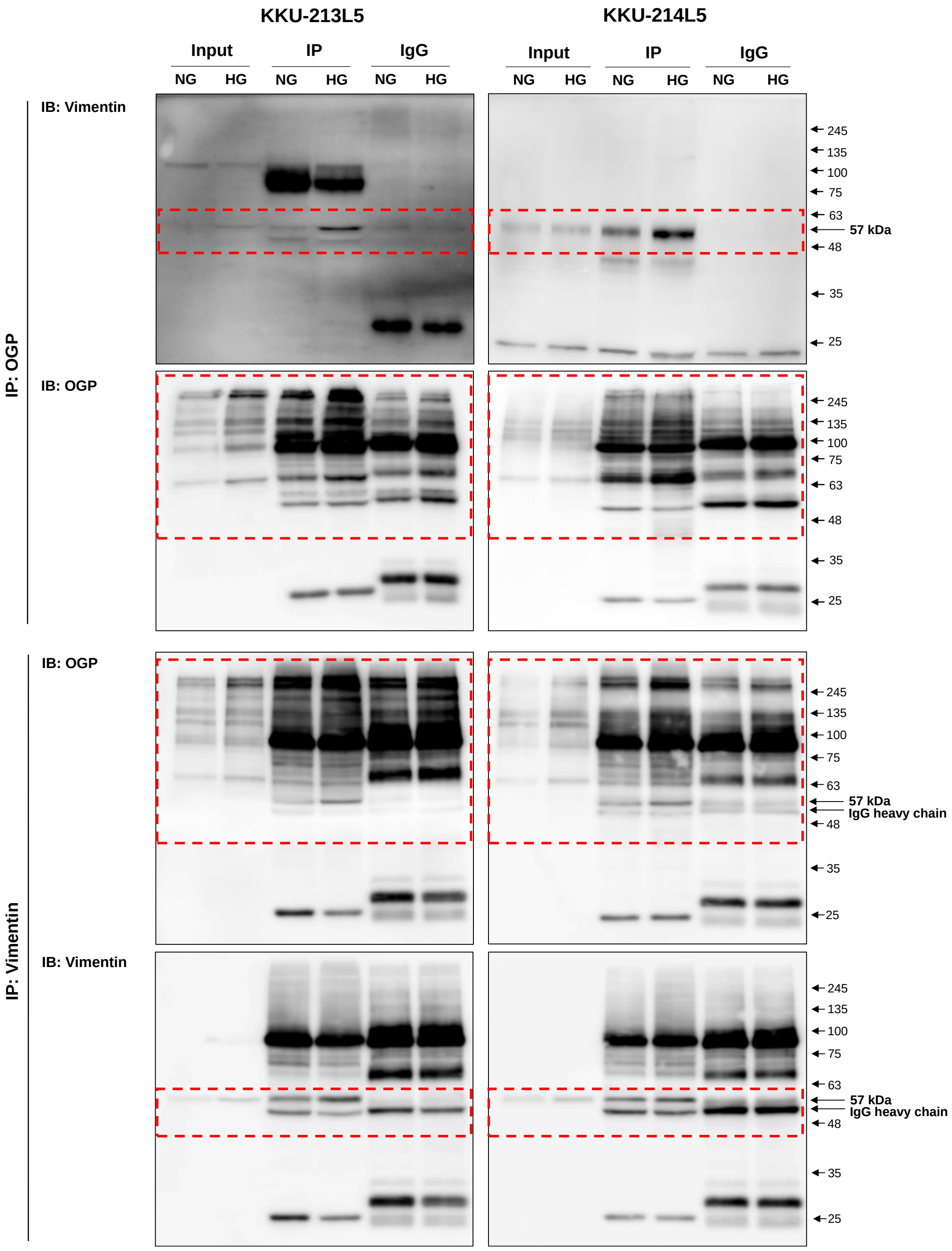


**Supplementary Figure S4. Full-length blots presented in Figure 3A.** Red boxes represented the selected areas.

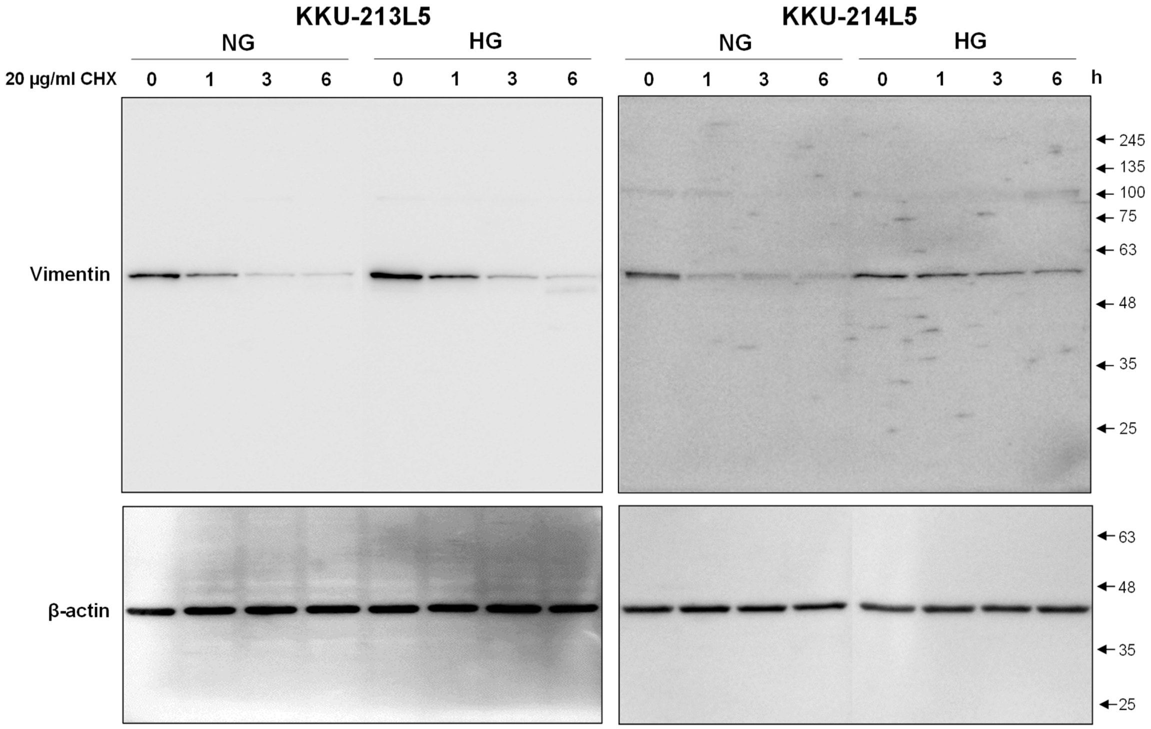


**Supplementary Figure S5. Full-length blots presented in Figure 3B.**

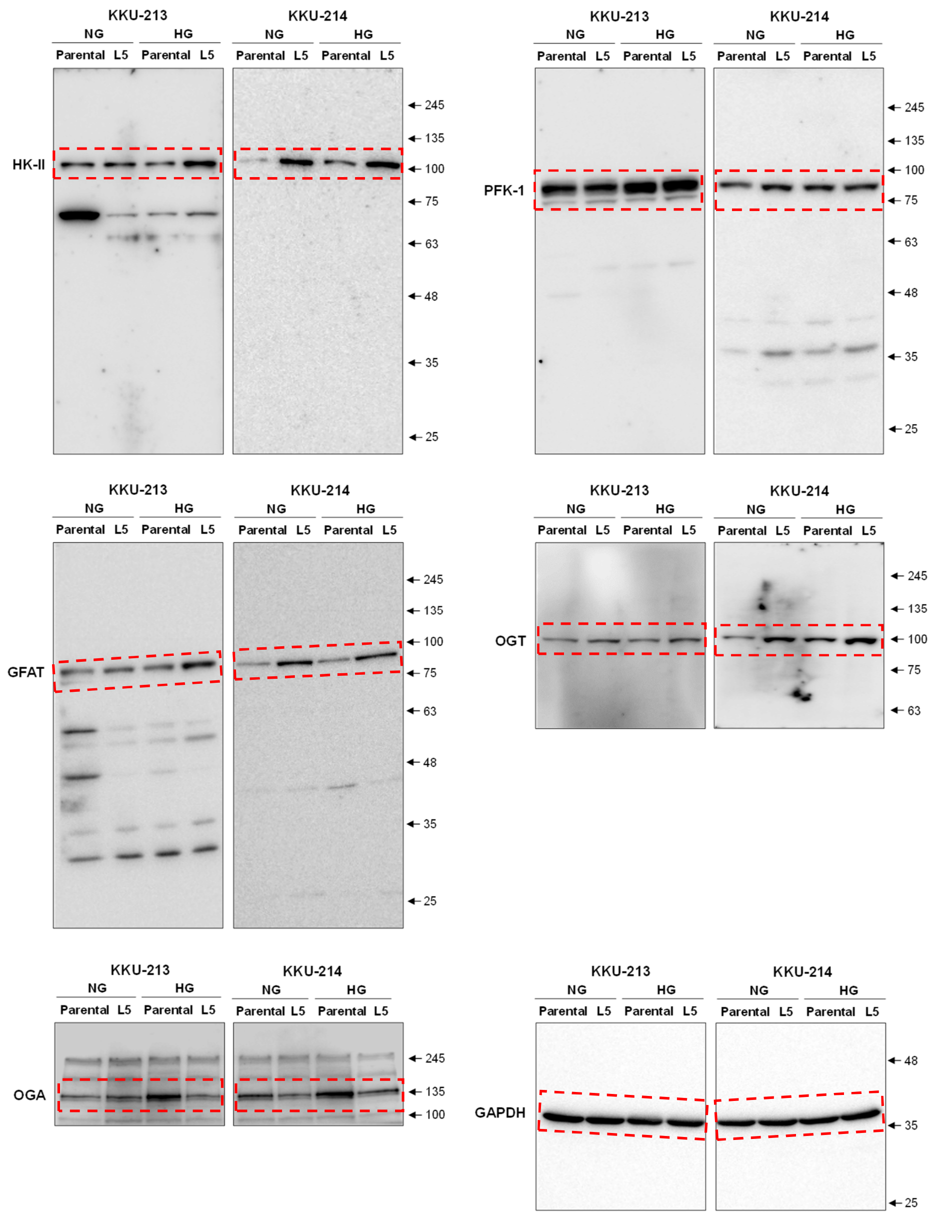


**Supplementary Figure S6. Full-length blots presented in Figure 4.** Red boxes represented the selected areas.

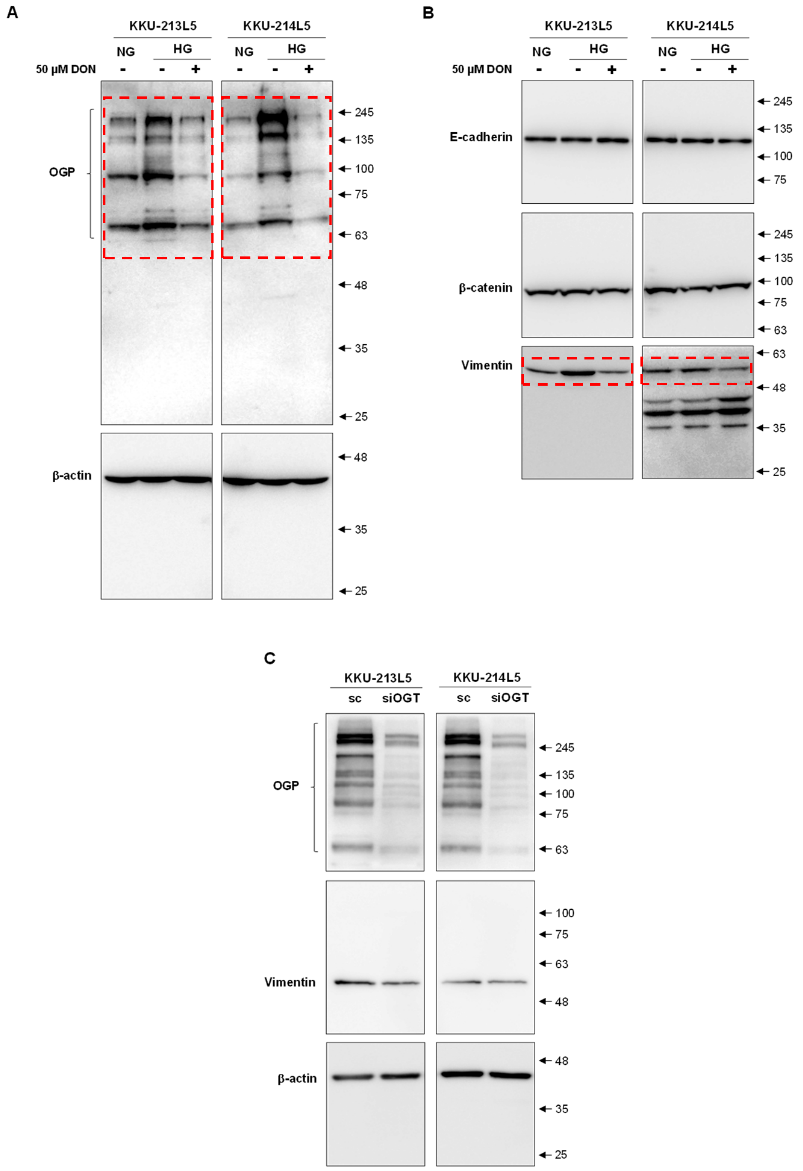


**Supplementary Figure S7. Full-length blots presented in Figure 5A (A), 5C (B), 5D (C).**

Red boxes represented the selected areas.

## Supplementary Table

### Supplementary Table S1. List of antibodies for western blot analysis

| Antibodies | Catalog # | Company | Dilution |
|---|---|---|---|
| Mouse monoclonal IgG anti-β-catenin (14) | 610153 | BD biosciences | 1:4,000 |
| Mouse monoclonal IgG anti-E-cadherin (36) | 610181 | BD biosciences | 1:1,000 |
| Rabbit monoclonal IgG anti-vimentin (D21H3) | 5741 | Cell Signaling Technology | 1:1,000 |
| Rabbit monoclonal IgG anti-slug (C19G7) | 9585 | Cell Signaling Technology | 1:1,000 |
| Mouse monoclonal IgG anti-O-GlcNAc (RL2) | MA 1-072 | Pierce Biotechnology | 1:1,000 |
| Mouse monoclonal IgG anti-β-actin | A5441 | Sigma-Aldrich | 1:10,000 |
| Goat polyclonal IgG anti-HK-II (C-14) | sc-6521 | Santa Cruz Biotechnology | 1:500 |
| Rabbit polyclonal IgG anti-PFK-1 (H-55) | sc-67028 | Santa Cruz Biotechnology | 1:1,000 |
| Rabbit polyclonal IgG anti-GFAT (H-49) | sc-134894 | Santa Cruz Biotechnology | 1:1,000 |
| Mouse monoclonal IgG anti-O-GlcNAc transferase (F12) | sc-74546 | Santa Cruz Biotechnology | 1:200 |
| Rabbit polyclonal IgG anti-O-GlcNAcase (OGA) | SAB4200267 | Sigma-Aldrich | 1:500 |
| Mouse monoclonal IgG anti-GAPDH | 92590 | Merck Millipore | 1:5,000 |
